# Association between Thrombophilia and the Post-Thrombotic Syndrome

**DOI:** 10.1155/2013/643036

**Published:** 2013-05-09

**Authors:** Anat Rabinovich, Susan R. Kahn

**Affiliations:** Center for Clinical Epidemiology, Jewish General Hospital, 3755 Côte Saint Catherine Room H420.1, Montreal, QC, Canada H3T 1E2

## Abstract

The post thrombotic syndrome (PTS) is a chronic condition that develops in 20%–40% of deep vein thrombosis (DVT) patients. While risk factors that predispose to the development of venous thromboembolism (VTE) are widely known, factors that influence the development of PTS after DVT have not been well elucidated. Over 10% of the general population is affected by one or more identifiable inherited thrombophilias which have been shown to underlie at least 1/3 of cases of VTE. The various thrombophilias are important risk factors for VTE, but it is unknown whether they also increase the risk for development of PTS. We performed a review of studies that have reported on the association between thrombophilia and the development of PTS in populations of patients with DVT and with chronic venous ulcers. Studies vary with regards to the definition of PTS, study design, follow-up period, and present conflicting results. Based on these results, the question of whether thrombophilia predisposes to the development of PTS remains unanswered.

## 1. Introduction

The post thrombotic syndrome (PTS) is a chronic condition that develops in 20%–40% of patients within 1-2 years after symptomatic deep venous thrombosis (DVT) [1]. There is no gold standard test for the diagnosis of PTS and the diagnosis is primarily based on clinical features. Patients with PTS experience pain, heaviness, and swelling in the affected limb which tend to be aggravated by standing or walking and improved with rest and recumbency. Edema, venous ectasia, hyperpigmentation, eczema, and varicose collateral veins may be apparent. In severe cases, leg ulceration can occur [2].

While hereditary and acquired risk factors that predispose to the development of venous thromboembolism (VTE) are widely known [3], factors that influence the development of PTS after DVT have not been well elucidated. One reason for the lack of understanding of PTS risk factors could be that most investigations have been limited to small retrospectively studied groups, with only a limited number of studies being prospectively undertaken over the long term [4–6]. Furthermore, great differences exist between studies regarding the definition of PTS, study design, follow-up period, and the possibility of bias when the effects of recurrent DVT cannot be clearly distinguished from PTS [7, 8]. Some papers use the clinical classification (clinical, aetiological, anatomical, and pathological (CEAP classification)) recommended by the International Consensus Committee on Chronic Venous Disease for evaluation of PTS [9], while others have used the Villalta scale [10, 11], as recommended by the International Society on Thrombosis and Haemostasis [12] or a variety of other scoring systems [13–15]. A comparison between studies is therefore difficult. 

Thrombophilia refers to an inherited or acquired predisposition to VTE. Over 10% of the general population is affected by one or more identifiable inherited thrombophilias which have been shown to underlie at least 1/3 of cases of VTE [16]. The inherited thrombophilias include factor V Leiden, prothrombin G20210A, antithrombin deficiency, protein C deficiency, protein S deficiency, and elevated coagulation factor VIII (FVIII), while acquired thrombophilias include the presence of antiphospholipid antibodies.

The prevalence of factor V mutation in patients with a history of DVT is approximately 18.8% [17, 18]. The relative risk for DVT is increased about 7-fold in heterozygote carriers and 40–80-fold in homozygote carriers [18]. The prothrombin 20210A allele is present in 7% of patients with VTE. The risk of VTE is 2- to 3-fold higher among heterozygotes [18]. The relative risk of DVT in patients with antithrombin deficiency is increased 5–10-fold [17]. Antithrombin deficiency can be identified in 1.9%–4.3% of patients with DVT [17, 18]. The prevalence of protein C deficiency is 3.7–4.8% in patients with DVT or recurrent DVT [17, 18]. The relative risk for a first DVT in these patients is increased 4–6.5-fold [19]. Patients with a history of DVT have a prevalence of protein S deficiency of 2.3%–4.3% [17, 18] and the relative risk for DVT is increased up to 10-fold [19]. Elevated FVIII level is also an independent inherited risk factor both for first and recurrent VTE [20–22]. The prevalence of antiphospholipid antibodies in patients with a history of DVT is not clear. Estimates range from 2% to 10% [23]. The relative risk for DVT is increased 3–10-fold for lupus anticoagulant, 0.7-fold for positive anticardiolipin antibodies and 2.4 fold for positive anti-b2 glycoprotein I antibodies [19]. 

Hence, the various thrombophilias are important risk factors for VTE, and PTS is a frequent complication of VTE, especially after recurrent DVT. The purpose of this review is to summarize available information on the association between thrombophilia and the development of PTS. 

## 2. Methods

We performed a computerized search of the literature (PubMed, EMBASE, Medline through PubMed, SCOPUS and Web of Science) from 1990 up to December 2012, using the search terms lupus coagulation inhibitor, lupus anticoagulant, antiphospholipid, factor VIII, antithrombin deficiency, protein S, protein C, prothrombin G20210A, factor V Leiden, thrombophilia, venous ulcers, postphlebitic syndrome, postthrombotic syndrome, venous stasis syndrome. We reviewed all articles that met the following criteria: studies enrolled adult patients with objectively diagnosed DVT or with chronic venous ulcers, studies described the incidence of PTS in the study population, enrolled patients had a thrombophilia workup during study participation, and data were presented on the prevalence of thrombophilia in the PTS and control subgroups.

For the purpose of this review, we divided studies into those involving DVT patients and those in chronic venous ulcer (CVU) patients.  Table 1 summarises the included studies in DVT patients and Table 2 in CVU patients. 

## 3. Studies in DVT Patients (Table 1)

In a seminal prospective cohort study by Prandoni et al. [24], 355 consecutive patients with a first episode of symptomatic DVT were followed up for 8 years. The aim was to determine the long-term risk for recurrent VTE and the incidence and severity of postthrombotic sequelae. Thrombophilia was found in 46 (13%) of patients (antithrombin deficiency, 10 patients; protein C deficiency, 9 patients; protein S deficiency, 13 patients; lupus-like anticoagulants, 14 patients). The presence of thrombophilia increased the risk for recurrent venous thromboembolism (hazard ratio 1.44, 95% CI, 1.02–2.01) but did not show a significant association with the risk of PTS. 

 In an expansion of Prandoni's original cohort [25], 528 consecutive symptomatic outpatients with a first episode of venography proven DVT were followed over an 8-year period. Thrombophilia was found in 69 (13.1%) patients; of these, 19 had protein S deficiency, 15 had antithrombin deficiency, 15 had protein C deficiency, and 20 had lupus-like anticoagulants. Thrombophilia increased the risk of recurrent venous thromboembolism (RR = 2.0). By contrast, there were again no significant associations between the occurrence of PTS and the presence of these coagulation abnormalities.

In another study by Prandoni et al. [26], a randomized controlled trial to evaluate the efficacy of compression elastic stockings for prevention of PTS in patients with proximal DVT, 180 consecutive patients with a first episode of symptomatic proximal DVT were included. Thrombophilia was present in 16 (17.8%) of the elastic stockings group (11 patients had factor V Leiden mutation, 1 had prothrombin G20210A mutation, 2 had deficiency of protein S, 1 had deficiency of protein C, and 1 had lupus-like anticoagulants), and in 17 (18.9%) of the control group (3 patients had factor V Leiden mutation, 5 had prothrombin G20210A mutation, 2 had deficiency of protein S, 1 had deficiency of protein C, and 6 had lupus-like anticoagulants). Thrombophilic status was not associated with development of PTS.

Schulman et al. [27], in an extended 10-year followup of a multicenter trial comparing secondary prophylaxis with vitamin K antagonists for 6 weeks versus 6 months that included 545 patients, found that neither carriage of factor V Leiden mutation or prothrombin G20210A polymorphism nor presence of cardiolipin antibodies was associated with an increased risk of developing PTS. 

Stain et al. prospectively followed 406 patients after a first symptomatic DVT for a median of 60 months, in order to establish risk factors of PTS [28]. Factor V Leiden (odds ratio (OR) 0.9, 95% CI 0.6–1.3), prothrombin G20210A (OR 0.8, 95% CI 0.4–1.7), or FVIII > 234 IU/dL (OR 2.0, 95% CI 0.8–5.1) did not confer an increased risk of PTS. 

Tick et al. determined the risk of PTS after DVT and assessed risk factors for PTS in 1668 consecutive patients who suffered a first DVT of the leg, in a follow-up study of the MEGA (Multiple Environmental and Genetic Assessment) study [29]. Factor V Leiden (relative risk (RR) 1.1, 95% CI 0.9–1.4) and the prothrombin G20210A mutation (RR 1.2, 95% CI 0.9–1.7) did not influence the risk of PTS. 

Biguzzi et al. [30] evaluated 51 women with at least one previous episode of symptomatic, objectively documented DVT before the age of 40. No correlation was found between PTS and the presence of coagulation abnormalities (deficiency of protein C, protein S, and antithrombin; factor V Leiden; lupus anticoagulant and hyperhomocysteinemia, all together present in 51% of patients).

In contrast to the above studies, Kahn et al., in a multicenter, prospective study of 145 patients with an unprovoked episode of proximal DVT, found that the presence of factor V Leiden or the prothrombin G20210A mutation was an independent predictor of both a lower risk (OR 0.33, 95% CI 0.15–0.73, *P* = 0.006) and reduced severity (1.6 point decrease (95% CI 0.06–3.2, *P* = 0.045) in Villalta score if thrombophilia was present) of PTS [31]. While this finding may have occurred by chance, it may have reflected true differences in the biology and natural history of thrombi that occur in patients with inherited thrombophilia compared with patients without inherited thrombophilia. For example, a review of seven primary studies reported that, among patients with VTE, those with factor V Leiden were half as likely to develop pulmonary embolism as those without factor V Leiden [42]. It has also been reported that patients with symptomatic DVT who have factor V Leiden have thrombi that are less extensive and less likely to involve the iliofemoral veins than those who do not have factor V Leiden [43]. Hence, there may be a tendency for factor V Leiden to predispose to smaller thrombi that are less likely to form emboli and to damage the venous valvular system (and thus induce PTS). However, a potential protective effect of thrombophilia was not confirmed in a later study by Kahn et al. [32]; among 387 outpatients and inpatients who received an objective diagnosis of acute symptomatic DVT, thrombophilia (factor V Leiden or prothrombin G20210A mutation) was not predictive of the development of PTS. 

Spiezia investigated 530 patients with DVT for the principal thrombophilias (antithrombin, proteins C and S deficiencies, lupus anticoagulant, factor V Leiden, and prothrombin G20210A mutation) [33]. Overall, there was no association between presence of thrombophilia and risk of PTS (adjusted hazard ratio (HR) 1.23; 95% CI 0.92–1.64; *P* = 0.15). However, by individual type of thrombophilia, compared to noncarriers, the adjusted HR for the development of PTS was 0.42 (95% CI, 0.20–0.88; *P* = 0.02) in carriers of factor V Leiden, 0.81 (0.36–1.37) in carriers of lupus anticoagulant, 0.96 (0.29–3.82) in carriers of protein C deficiency, 1.08 (0.29–2.70) in carriers of protein S deficiency, and 1.33 (0.68–2.58) in carriers of prothrombin G20210A mutation. Neither of the two patients with antithrombin deficiency developed PTS. Hence, factor V Leiden again appeared to protect against development of PTS, perhaps, according to the authors, because isolated involvement of the popliteal vein occurred more frequently in carriers of factor V Leiden (50/85, 58.8%) than in non-carriers (193/445, 43.4%; *P* = 0.012). In the subgroup of 85 patients with factor V Leiden, PTS developed in 11 of 50 patients (22.0%) with involvement of the popliteal vein only and in 18 of 35 patients (51.4%; *P* = 0.01) with more proximal thrombosis. 

 Bittar et al., in a recent study [34], evaluated FVIII levels in patients with DVT of the lower limbs. In 2004, in a first assessment, FVIII levels were evaluated in 230 patients and in 230 matched controls. In 2011, 55 patients from the initial cohort who originally presented with FVIII levels above the 90th percentile, were recruited for a second assessment of FVIII activity and compared to 74 healthy controls. The study analyzed the relationship between FVIII levels and subsequent PTS. At the first assessment, a median of 3 years after venous thrombosis, this cohort showed higher plasma FVIII when compared to controls. On the second assessment, a median of 10 years after the index DVT, FVIII levels were significantly lower when compared to the initial assessment (*P* < 0.001), with a mean reduction of 33%. However, FVIII levels were still significantly higher in patients compared to controls (*P* < 0.001). Patients with severe PTS showed increased levels of FVIII compared to patients with moderate or absent PTS (*P* < 0.001). As Von Willebrand Factor (VWF) plays a critical role in regulating plasma FVIII levels [44], a possible explanation is that, in severe PTS, vascular injury with associated increased endothelial cell secretion of VWF could contribute to the increased plasma FVIII levels found in those patients. 

Based on the above mentioned studies, the risk of PTS in carriers of the most common thrombophilic abnormalities who develop an episode of DVT, does not seem to exceed that expected in non-carriers, and may even be diminished by the carriage of factor V Leiden, perhaps because of the more distal location of the thrombotic episode in carriers as compared with non-carriers of this abnormality [32, 45]. Overall, the role of inherited thrombophilia with regard to the risk of PTS after DVT is still not well established.

## 4. Studies in Patients with Chronic Venous Ulcers

Severe PTS can lead to the development of leg ulcers. The available literature suggests that between 25% and 75% of patients with CVU have postthrombotic disease on the basis of a history of DVT or evidence of postphlebitic changes in the deep venous system on investigation [46]. However, these estimates vary widely according to the population studied and the thoroughness with which history and investigative evidence of prior DVT are sought. This review will encompass only studies in CVU patients that examined the relationship between thrombophilia and PTS (i.e., CVU in a patient with previous DVT). The studies that exist are small and often methodologically flawed, and give a conflicting picture. Table 2 summarises the studies included in this review. 

Zutt et al., in a cohort study of 310 patients [35], stratified into patients with and without PTS, as determined by sonographic and/or phlebographic findings, found that protein S deficiency (*P* = 0.035) and factor V Leiden (*P* = 0.003) were significantly more prevalent in the PTS group. 

Hafner et al. [36] determined the prevalence of the factor V Leiden mutation in 73 patients with postthrombotic and nonpostthrombotic venous ulcers. PTS was identified as the cause of 42 (58%) of venous ulcers. In postthrombotic ulcers, the prevalence of the factor V Leiden mutation was 38% versus 16% in non-postthrombotic venous ulcers, corresponding to an odds ratio of 3.2.

Wiszniewski et al. [37] showed that one-third of patients with CVU have at least one inherited thrombophilia. Moreover, all patients with inherited thrombophilia in this study experienced at least one previous DVT episode. In 94% of patients with CVU and thrombophilia, DVT was recurrent, and in 88% of them, both recurrent DVT and recurrent CVU were observed. CVU also persisted longer when compared to patients with CVU and no thrombophilia, despite similar management. Hence, this study shows that thrombophilia is a common finding in CVU patients as a whole. Although a precise assessment cannot be given on the basis of this study, it might also suggest that thrombophilia is more common in postthrombotic ulcers. 

Maessen-Visch et al. [38] found 21 (23%) factor V Leiden mutation carriers among 92 patients. Ninety-one percent of patients with CVU and factor V Leiden had a clinical history of DVT (i.e., PTS-related leg ulcers) as compared with 48% of those without the mutation (*P* = 0.002). However, not all patients had objectively documented prior DVT and did not undergo investigation with venography or duplex ultrasound scan.

Gaber et al. determined the prevalence of APC resistance in 100 patients [39]. The underlying cause for venous ulcers was classified using Doppler ultrasound, duplex scanning, and light reflex rheography or phlebography. APC resistance was detected in 36% of patients with postthrombotic leg ulcers and 6% of patients with ulcers caused by primary varicosis. 

MacKenzie et al. [40] found that among 88 patients, 36% had either a history or duplex scan evidence suggestive of previous DVT. Thrombophilia was not significantly related to previous DVT, deep reflux, or disease severity.

Ribeaudeau et al. [41] found APC resistance in 4 (11%) of 35 patients, but the factor V Leiden mutation was confirmed in only 1 of these 4 cases (3%). Forty percent of the patients had a history of DVT. There was no difference in DVT rates between those with and without thrombophilia. 

How thrombophilia might influence the pathophysiology of CVU is unclear. Thrombophilia may predispose to the development of superficial and deep lower limb venous reflux as a result of macrovascular thromboembolic disease, with subsequent venous hypertension, skin changes, and ultimately venous ulceration. Microthromboses in venules or small arteries with subsequent ischemic skin breakdown are another possibility [35, 47, 48].

To summarize, as in DVT patients, it is still unclear whether thrombophilia influences the risk of developing postthrombotic ulcers, a severe form of PTS. The results of these small studies are conflicting, and there is uncertainty about the diagnosis of PTS in most studies. Moreover, thrombophilia could have a direct influence on CVU development, not necessarily through increased rates of DVT or PTS, and this potential pathway needs to be addressed in further studies.

## 5. Conclusion 

We performed a review of studies that have reported on the association between thrombophilia and the development of PTS in populations of patients with DVT and with CVU. Based on the conflicting evidence presented, there is still no clear cut answer to the question of whether thrombophilia predisposes to the development of PTS. Hence, it is currently not possible to estimate an individual DVT patient's risk of PTS based on whether or not thrombophilia is present. Larger prospective studies that use a strict definition of PTS are needed to resolve this issue. 

## Figures and Tables

**Table 1:** Included studies in deep vein thrombosis patients.

Reference	Author/year	Type of patient	Number of patients included	Thrombophilias assessed	*N* (%) with PTS	Criteria for PTS diagnosis
[24]	Prandoni et al., 1996	DVT	355	LAC, PC, PS, AT	99 (28)	Villalta
[25]	Prandoni et al., 1997	DVT	528	LAC, PC, PS, AT	119 (22.5)	Villalta
[26]	Prandoni et al., 2004	DVT	180	FVL, PTM, LAC, PC, PS	23 (25.6) patients 44 (48.9) controls	Villalta
[27]	Schulman et al., 2006	DVT	545	FVL, PTM, APL	306 (56.3)	CEAP
[28]	Stain et al., 2005	DVT	406	FVIII, FVL, PTM	176 (43.3)	CEAP
[29]	Tick et al., 2008	DVT	1668	FVL, PTM	417 (25)	Modified Villalta
[30]	Biguzzi et al., 1998	DVT	51	FVL, LAC, PC, PS, AT	32 (63)	CEAP
[31]	Kahn et al., 2005	DVT	145	FVL, PTM	54 (37.2)	Villalta
[32]	Kahn et al., 2008	DVT	387	FVL, PTM	102 (40)	Villalta
[33]	Spiezia et al., 2010	DVT	530	FVL, PTM, LAC, PC, PS, AT	172 (32.5)	Villalta
[34]	Bittar et al., 2012	DVT	55	FVIII	31 (56.3)	CEAP

FVL: factor V Leiden, PTM: prothrombin G20210A, APC res: activated protein C resistance, LAC: lupus anticoagulant, APL: antiphospholipid antibodies, PC: protein C deficiency, PS: protein S deficiency, AT: antithrombin deficiency, FVIII: coagulation factor VIII, DVT: deep vein thrombosis, CVU: chronic venous ulcer, PTS: postthrombotic syndrome, and CEAP: clinical, aetiological, anatomical, and pathological classification.

**Table 2:** Included studies in chronic venous ulcer patients.

Reference	Author/year	Number of patients included	Criteria for PTS diagnosis	*N* (%) with PTS	Number with thrombophilia	Association between thrombophilia and PTS
[35]	Zutt et al., 2011	310	Duplex Doppler ultrasound and/or venography	142 (45.8)	59 FVL	PTS positive 42/127, PTS negative 17/128, *P* = 0.001
					57 APC res	PTS positive 40/127, PTS negative 17/128, *P* = 0.061
					8 PTM	PTS positive 5/125, PTS negative 3/161, *P* = 0.411
					2 LAC	PTS positive 2/120, PTS negative 0/144, *P* = 0.121
					75 APL	ACL-PTS positive 15/112, PTS negative 29/124, *P* = 0.493 Ab2GI-PTS positive 17/110, PTS negative 14/114, *P* = 0.581
					22 PC	PTS positive 15/105, PTS negative 7/112, *P* = 0.078
					35 PS	PTS positive 23/95, PTS negative 12/108, *P* = 0.001
					34 AT	PTS positive 16/139, PTS negative 18/160, *P* = 0.751

[36]	Hafner et al., 2001	73	Duplex Doppler ultrasound and history of DVT	42 (58)	21 FVL	PTS positive 16/42, PTS negative 5/31, OR 3.2 95% CI 1–10, *P* = 0.07

[37]	Wiszniewski et al., 2011	110	Duplex Doppler ultrasound and history of DVT	64 (58.2)	20 FVL 2 PTM 8 PS 7 PC 3 AT 5 LAC 12 ACL	1/3 of patients with CVU have ≥1 thrombophilia All patients with thrombophilia experienced ≥1 previous DVT In 94% of patients with CVU and thrombophilia, DVT was recurrent, and in 88% of them, both recurrent DVT and recurrent CVU were observed CVU persisted longer compared to patients with CVU and no thrombophilia

[38]	Maessen-Visch et al., 1999	92	Light reflex rheography and history of DVT	53 (57.6)	21 FVL	PTS positive 19/53, PTS negative 2/39, *P* = 0.002

[39]	Gaber et al., 2001	100	Duplex Doppler ultrasound, light reflex rheography or phlebography	53 (53)	22 APC res	PTS positive 19/53, PTS negative 3/47, OR 8.2 95% CI 2.55–26.33

[40]	MacKenzie et al., 2002	88	Duplex Doppler ultrasound and/or history of DVT	22 (25)	3 FVL	PTS positive 2/22, PTS negative 1/66, *P* = NS
					14 APC res	PTS positive 5/22, PTS negative 9/66, *P* = NS
					11 PTM	PTS positive 3/22, PTS negative 8/66, *P* = NS
					6 PS	PTS positive 2/22, PTS negative 4/66, *P* = NS
					5 PC	PTS positive 2/22, PTS negative 3/66, *P* = NS
					4 AT	PTS positive 3/22, PTS negative 1/66, *P* = 0.02
					8 LAC	PTS positive 1/22, PTS negative 7/66, *P* = NS
					12 ACL	PTS positive 6/22, PTS negative 6/66, *P* = 0.03

[41]	Ribeaudeau et al., 1999	35	Duplex Doppler ultrasound and/or history of DVT	14 (40)	1 FVL	PTS positive 1/10, PTS negative 0/20
					4 APC res	PTS positive 1/14, PTS negative 3/21
					2 PTM	PTS positive 1/10, PTS negative 1/20
					3 APL	PTS positive 1/12, PTS negative 2/18

FVL: factor V Leiden, PTM: prothrombin G20210A, APC res: activated protein C resistance, LAC: lupus anticoagulant, APL: antiphospholipid antibodies, ACL: Anticardiolipin antibodies, PC: protein C deficiency, PS: protein S deficiency, AT: antithrombin deficiency, FVIII: coagulation factor VIII, DVT: deep vein thrombosis, CVU: chronic venous ulcer, PTS: post thrombotic syndrome, OR: odds ratio, CI: confidence interval, and NS: nonsignificant.
